# A novel clinical diagnostic marker predicting the relationship between visceral adiposity and renal function evaluated by estimated glomerular filtration rate (eGFR) in the Chinese physical examination population

**DOI:** 10.1186/s12944-023-01783-6

**Published:** 2023-03-04

**Authors:** Yanping Xu, Xin Yi Wang, Haiping Liu, Dongzhen Jin, Xiaoxiao Song, Shengyao Wang, Xinhe Zhou, Mengte Shi, Chao Zheng, Xiaoyou Su

**Affiliations:** 1 Department of Endocrinology, The Second Affiliated Hospital and Yuying Children’s Hospital of Wenzhou Medical University, Zhejiang, China; 2 Department of Nephrology, The Second Affiliated Hospital and Yuying Children’s Hospital of Wenzhou Medical University, Zhejiang, China; 3 Division of Epidemiology and Health Statistics, Department of Preventive Medicine, School of Public Health & Management, Wenzhou Medical University, Zhejiang, China; 4 Department of Endocrinology, The Second Affiliated Hospital Zhejiang University School of Medicine, Hangzhou, Zhejiang China

**Keywords:** Chronic kidney disease, Chinese visceral obesity index, Visceral obesity, Screening

## Abstract

**Background and aims:**

The effect of body fat deposition on the kidney has received increasing attention. The Chinese visceral adiposity index (CVAI) is an important indicator of recent research. The purpose of this study was to explore the predictive value of CVAI and other organ obesity indicators in predicting CKD in Chinese residents.

**Methods:**

A retrospective cross-sectional study of 5355 subjects was performed. First, the study utilized locally estimated scatterplot smoothing to describe the dose–response relationship between the estimated glomerular filtration rate (eGFR) and CVAI. The L1-penalized least absolute shrinkage and selection operator (LASSO) regression algorithm was used for covariation screening, and the correlation between CVAI and eGFR was quantified using multiple logistic regression. At the same time, the diagnostic efficiency of CVAI and other obesity indicators was evaluated by ROC curve analysis.

**Results:**

CVAI and eGFR were negatively correlated. Using group one as the control, an odds ratio (OR) was calculated to quantify CVAI quartiles (ORs of Q2, Q3, and Q4 were 2.21, 2.99, and 4.42, respectively; *P* for trend < 0.001). CVAI had the maximum area under the ROC curve compared with other obesity indicators, especially in the female population (AUC: 0.74, 95% *CI*: 0.71–0.76).

**Conclusions:**

CVAI is closely linked to renal function decline and has certain reference value for the screening of CKD patients, particularly in women.

**Supplementary Information:**

The online version contains supplementary material available at 10.1186/s12944-023-01783-6.

## Introduction

Chronic kidney disease (CKD) is a major public problem that endangers human life and health [1]. The prevalence of CKD in China is as high as 10.8%, and 132 million people are expected to suffer from CKD [2]. The rapid rise in CKD prevalence had a significant negative effect on China. The cost of dialysis treatment for just one patient is approximately US $14,300, resulting in a heavy economic burden and reducing the quality of life [3]. Furthermore, CKD can promote the risk for hospitalization, cardiovascular disease, cognitive dysfunction, and death from any cause, as well as significantly impacting the prognosis of hypertension, diabetes, and cardiovascular disease [4]. Therefore, early detection and timely treatment of CKD are beneficial for controlling and delaying the occurrence of cardiovascular disease and end-stage renal disease (ESRD).

Epidemiological surveys have proven that obesity is a major risk factor for CKD. It is not only a single cause of kidney damage but also aggravates disease progression to ESRD and accelerates kidney damage caused by other diseases [5–7]. However, even people with the same body mass index (BMI) may have different risks for metabolic and cardiovascular disease [8]. As a result, the effect of human fat distribution on CKD remains unknown. Previous research has shown that WC (as the representative for abdominal obesity) is more strongly associated with CKD than BMI (as the representative for general obesity). However, WC does not account for the effect of height on risk, so the waist-hip ratio (WHR) was introduced as an alternative to WC. However, the biological complexity of obesity was not accounted for [9–11].

Visceral fat has been proven to promote metabolic disorders [12]. The gold standard used clinically to evaluate visceral fat content is magnetic resonance imaging (MRI) or computed tomography (CT) [13, 14]. However, due to the high price of the machine and side effects such as ionizing radiation, the application of visceral fat evaluation in screening a large sample population is limited. The simple calculation of the visceral fat index (VAI) correlates well with visceral fat measurement by imaging and is also significantly correlated with the incidence of CKD; however, since the fat distribution characteristics of Asians differ from those of Europeans and Americans, it is not entirely applicable to Asians [15, 16]. Xia proposed a new index to assess visceral fat, the Chinese visceral obesity index (CVAI), which combines VAI and incorporates fat distribution characteristics in Asians [17]. Currently, the correlation between CKD and CVAI has not been reported. Therefore, the purpose of this study is to clarify the relationship of CKD with CVAI as well as five other anthropometric indicators—BMI, WC, waist-hip ratio (WHR), VAI, and lipid accumulation production (LAP)—using data extracted from community-based routine physical examination records in a Chinese population. Furthermore, we would like to obtain appropriate cutoff points for those obesity indicators when diagnosing CKD.

## Methods

### Study design and participants

Participants were enrolled in physical examinations at the Physical Examination Center of Zhejiang University’s Second Affiliated Hospital from January 1, 2020, to December 31, 2020. People with missing CVAI data, kidney stones, positive urine routine proteinuria, positive urinary occult blood, and urinary nitrate to remove urinary tract infections were excluded from this study. Moreover, this study excluded people with missing calculated values of eGFR and eGFR > ^120 mL/min/1.73 m2^. Finally, a total of 5355 individuals were included, with 1858 cases (eGFR < ^90 mL/min/1.73 m2^) and 3497 controls (^90 mL/min/1.73 m2^ ≤ eGFR ≤^120 mL/min/ 1.73 m2^) (Fig. S1). The Ethics Committee of our hospital approved the study. All patients signed to confirm.

### Definition of renal function injury

Renal function injury was defined by calculated eGFR values from the CKD-EPI combined formula. The subjects were divided into two groups: eGFR < 90 ml/min/1.73 m^2^ (renal function injury group) ^and 90 ml/min/1.73 m2^ ≤ eGFR ≤^120 ml/min/1.73 m2^ (healthy group).

### Data collection

Data on demographics and clinical information of participants, such as age, sex, and others, were obtained from interviews or hospital medical records. Blood and urine samples were collected after fasting for at least 8 hours.

### Physical examination

This study uses a calibrated digital platform scale to measure weight to 0.1 kg. A freestanding rangefinder with an accuracy of 0.5 cm is used to measure the standing height. BMI was calculated by dividing weight in kilograms by height in meters squared. WC was measured at the level of the umbilical cord in a standing population with an accuracy of 0.1 cm [18]. WHR was calculated using WC/HC, and hip circumference (HC) was measured accurately to 0.5 cm at the widest hip. After resting for at least 5 minutes, blood pressure (BP) was measured while seated with a calibrated mercury sphygmomanometer.

### Laboratory data collection and calculation formula

All samples were sent to the testing center laboratory of Zhejiang University’s Second Affiliated Hospital, and biochemical analysis of blood and urine was completed within 2 hours. This study recorded available laboratory data, including hemoglobin, fasting blood glucose, glycosylated hemoglobin, triglyceride, total cholesterol, high-density lipoprotein, low-density lipoprotein, carcinoembryonic antigen, CA211, SCC, uric acid, urinary specific gravity, urinary occult blood, urinary nitrite, and urinary protein. Lydman enzymatic and turbidimetric assays were used to detect serum creatinine and cystatin. All data were measured by well-trained and experienced technicians in the Second Affiliated Hospital of Zhejiang University School of Medicine central laboratory using standard reagents and automatic biochemical analyzers in strict accordance with standard technical procedures. Participants were analyzed for eGFR using the CKD-EPI equation as the grouping basis.

The LAP, VAI, and CVAI were calculated as follows:

Males:

$${\displaystyle \begin{array}{c}\textrm{LAP}=\left[\textrm{WC}\;\left(\textrm{cm}\right)\hbox{-} 65\right]\times \textrm{TG}\;\left(\textrm{mmol}/\textrm{L}\right);\\ {}\textrm{VAI}=\textrm{WC}\;\left(\textrm{cm}\right)/\left[39.68+1.88\times \textrm{BMI}\;\left(\textrm{kg}/{\textrm{m}}^2\right)\right]\\ {}\textrm{CVAI}=\hbox{-} 267.93+0.68\times \textrm{age}+0.03\times \textrm{BMI}\;\left(\textrm{kg}/{\textrm{m}}^2\right)+4.00\times \textrm{WC}\;\left(\textrm{cm}\right)+22.0\times \log\;10\textrm{TG}\left(\textrm{mmol}/\textrm{L}\right)\hbox{-} 16.32\times \textrm{HDL-C}\;\left(\textrm{mmol}/\textrm{L}\right);\end{array}}\times \left[\textrm{TG}\;\left(\textrm{mmol}/\textrm{L}\right)/1.03\right]\times \left[1.31/\textrm{HDL-C}\;\left(\textrm{mmol}/\textrm{L}\right)\right];$$

Females:

$${\displaystyle \begin{array}{c}\textrm{LAP}=\left[\textrm{WC}\;\left(\textrm{cm}\right)\hbox{-} 58\right]\times \textrm{TG}\;\left(\textrm{mmol}/\textrm{L}\right);\\ {}\textrm{VAI}=\textrm{WC}\;\left(\textrm{cm}\right)/\left[36.58+1.89\times \textrm{BMI}\;\left(\textrm{kg}/{\textrm{m}}^2\right)\right]\\ {}\textrm{CVAI}=\hbox{-} 187.32+1.71\times \textrm{age}+4.32\times \textrm{BMI}\;\left(\textrm{kg}/{\textrm{m}}^2\right)+1.12\times \textrm{WC}\;\left(\textrm{cm}\right)+39.76\times \log\;10\textrm{TG}\left(\textrm{mmol}/\textrm{L}\right)\hbox{-} 11.66\times \textrm{HDL-C}\;\left(\textrm{mmol}/\textrm{L}\right);\end{array}}\times \left[\textrm{TG}\;\left(\textrm{mmol}/\textrm{L}\right)/0.81\right]\times \left[1.52/\textrm{HDL-C}\;\left(\textrm{mmol}/\textrm{L}\right)\right];$$

### Statistical analysis

All data and statistical analyses were performed using Stata/MP15.1 for Windows and version R 4.0.4 for Windows. *P* < 0.05 was considered a significant difference. The qualitative data are expressed as frequencies (percentages), while the quantitative data were first analyzed using the normality test, and normally distributed continuous variables are presented as the mean ± standard deviation (X ± SD) for statistical processing. The data exhibiting a non-normal distribution are described by the median. Two different sampling t tests were used to quantify the normal distribution. In contrast, the rank sum test is used for quantitative data that are not normally distributed. Fisher’s exact test or the chi-square test probability method was used to compare and analyze the qualitative data. To ensure the rigor of the results, median filling of continuous variables and modal filling of classified variables are carried out. A sensitivity analysis was performed to compare the differences before and after filling to evaluate the effect of filling (Table S1). The study first used the LOESS curve to describe the dose–response relationship between CVAI and eGFR. To avoid potential multicollinearity and overfitting as much as possible, a variety of effective measures were taken in the following multivariate analysis. 1) When comprehensively quantifying the association of CVAI and eGFR, covariates should be adjusted. L1-Penalized Least Absolute Shrinkage and Selection Operator (LASSO) regression was used. Depending on this algorithm, the absolute magnitude of each coefficient in the model would be separately penalized according to the value of λ. As the penalty increases, the estimates of some weaker covariates would gradually shrink toward zero. In the end, only the strongest predictors, whose effects should be considered only in extreme cases, remained in the model. 2) The impacts of variables known to be associated with renal function were carefully evaluated to determine whether they should be included in the covariates. 3) The study excluded age, sex, creatinine, and cystatin C from the calibration model because they were used to calculate eGFR. To comprehensively quantify the real relationship between the presence of CVAI and eGFR, a variety of multiple logistic regression models were fitted in the following ways: with CVAI as continuous variables [scaled to interquartile range (IQR)] and categorical variables (quartiles) with adjusting for the impacts due to the covariates. At the same time, associated linear trend tests were performed. The ROC curve was used to evaluate the accuracy of each obesity index in diagnosing renal function damage, and the Youden index was used to determine the best diagnostic cutoff point.

## Results

### Population characteristics

Table 1 shows the demographic and clinical characteristics of the participants. Among the 5355 people enrolled in the study (64.54% of whom were male), renal function decreased (74.4%, male) in 34.70%, with a slight decrease in renal function as the main feature. The mean CVAI values in the control and case groups were 92.77 (62.50, 122.02) and 115.72 (88.96, 140.25), respectively, indicating that individuals with decreased renal function had significantly higher CVAI values than those in the control group. The calculated CVAI value and the decline in renal function had a significant correlation. Table 1 also reveals significant differences in baseline levels of certain measures between the two groups, indicating that age, body mass index, sex, blood pressure, glycolipid metabolism, hemoglobin, carcinoembryonic antigen, and other indicators may be related to renal function damage. In addition, the results of the study showed that there was no significant difference in cholesterol levels.

**Table 1:** Clinical and demographic characteristics of the study population

Variables	90 ≤ eGFR ≤ 120	eGFR < 90	***P value***
Age (years)	48.0 (42.0,54.0)	57.0 (51.0,63.0)	< 0.001
Sex			< 0.001
Male	2074 (59.3)	1382 (74.4)	
Female	1423 (40.7)	476 (25.6)	
Height (cm)	165.5 (159.5171.0)	167.0 (161.0,171.5)	< 0.001
Weight (kg)	65.9 (57.6,74.4)	69.1 (61.3,77.4)	< 0.001
BMI (kg/m2)	24.0 (21.9,26.2)	25.0 (22.9,27.0)	< 0.001
Pulse	76 (69,83)	75 (68,83)	< 0.001
DBP (mmHg)	70 (62.,78)	72 (64,80)	< 0.001
SBP (mmHg)	119 (108,131)	126 (114,137)	< 0.001
FPG (mmol/L)	4.83 (4.52,5.25)	4.93 (4.60,5.39)	< 0.001
Hemoglobin(g/L)	146.00 (135.00,157.00)	149.00 (139.00,159.00)	< 0.001
HbA1c, %	5.60 (5.40,5.90)	5.80 (5.50,6.10)	< 0.001
TC (mmol/L)	5.11 (4.50,5.77)	5.16 (4.51,5.88)	0.201
TG (mmol/L)	1.30 (0.90,1.93)	1.44 (1.04,2.07)	< 0.001
HDL (mmol/L)	1.29 (1.11,1.50)	1.22 (1.06,1.41)	< 0.001
LDL (mmol/L)	2.73 (2.26,3.22)	2.84 (2.37,3.33)	< 0.001
Hs-CRP (mg/L)	0.80 (0.40,1.40)	1.00 (0.50,1.90)	< 0.001
UA (μmol/L)	327.00 (269.00,390.00)	368.00 (313.00,427.00)	< 0.001
BUN (mmol/L)	4.75 (4.08,5.52)	5.28 (4.58,6.04)	< 0.001
SG	1.02 (1.02,1.03)	1.02 (1.02,1.02)	< 0.001
CA211(ug/ml)	1.50 (1.00,2.00)	1.70 (1.20,2.40)	< 0.001
CEA (ng/ml)	1.70 (1.10,2.50)	2.10 (1.40,3.00)	< 0.001
SCC (ug/L)	0.80 (0.60,1.00)	0.90 (0.70,1.10)	< 0.001
Albumin(g/L)	42.50 (40.70,44.10)	42.00 (40.40,43.50)	< 0.001
TT3(ng/ml)	1.48 (1.34,1.63)	1.54 (1.40,1.69)	< 0.001
WC (cm)	83.0 (76.0,89.0)	87.0 (81.0,93.0)	< 0.001
LAP	27.06 (15.10,45.24)	33.89 (20.74,53.20)	< 0.001
CVAI	92.77 (62.50,122.02)	115.72 (88.96,140.25)	< 0.001
VAI	1.46 (0.96,2.31)	1.65 (1.09,2.53)	< 0.001
WHR	0.90 (0.85,0.94)	0.93 (0.89,0.97)	< 0.001

Control: 90 ≤ eGFR≤120 mL/min/1.^73 m2^; Case: eGFR< 90 mL/min/1.^73 m2^; Data were presented as the mean ± standard deviation for normal or similar normal distributed variables, number (percentage) for categorical variables, or median (25th,75th percentiles) for variables having skewed distribution. The comparison of normally distributed variables between cases and controls was tested by t tests, and skewed distributed variables were tested by Mann–Whitney U tests

*Abbreviation: BMI* body mass index, *DBP* diastolic blood pressure, *SBP* systolic blood pressure, *FPG* fasting plasma glucose, *HbA1c* glycosylated hemoglobin, *TG* triglyceride, *TC* cholestenone, *HDL* high-density lipoprotein, *LDL* low-density lipoprotein, *hs-CRP* hypersensitive C-reactive protein, *UA* uric acid, *BUN* blood urea nitrogen, *SG* specific gravity, *CA211* carbohydrate antigen 211, *CEA* carcinoembryonic antigen, *SCC* squamous cell carcinoma, *TT3* total triiodothyronine

### The correlation between CVAI and renal function

**Fig. 1:**
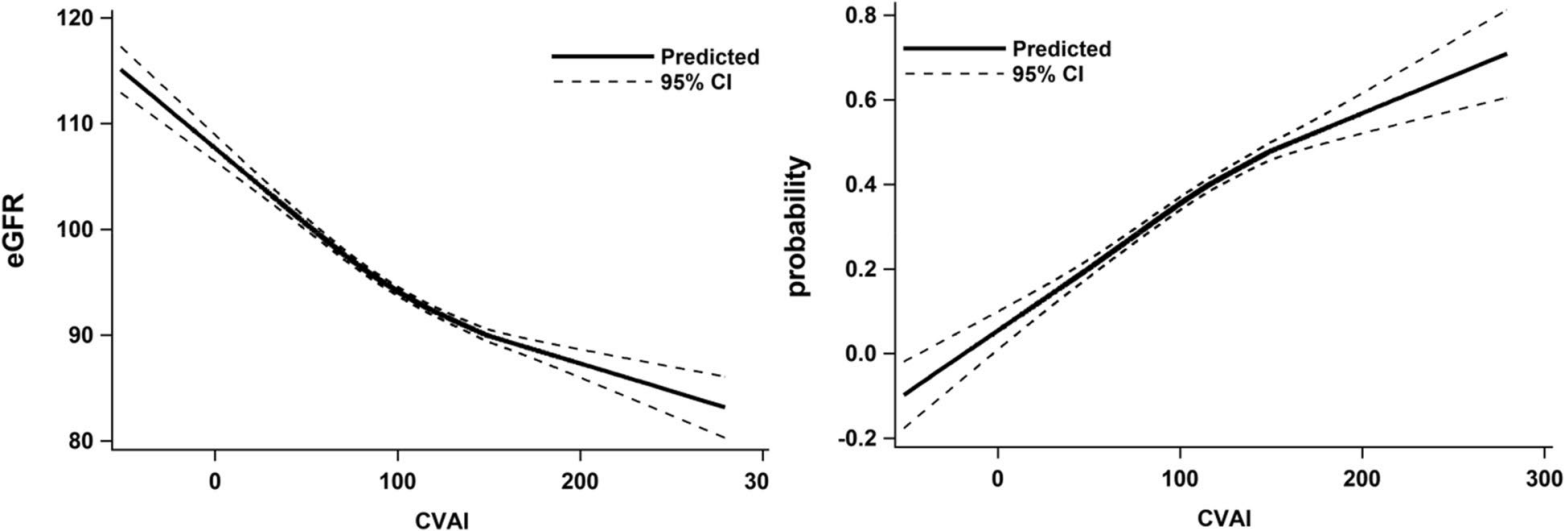
Correlation analysis between CVAI and eGFR

The correlation between CVAI and eGFR (Fig. 1) was analyzed using a locally estimated scatterplot smoothing (LOESS) curve. The findings revealed that as CVAI increased, eGFR decreased significantly, and the possibility of renal function injury increased significantly, indicating a negative correlation between CVAI and renal function level.

To further confirm the relationship between CVAI and renal function, the researchers divided 5355 participants into four levels based on CVAI calculated values: ≤ 70.59 (Q1), 70.60–101.93 (Q2), 101.94–130.23 (Q3), and 130.24–273.94 (Q4). The incidence rates of renal failure in the four groups were 17.90, 32.50, 39.40, and 49.00%, respectively. Compared with Q1, the odds ratio [*OR*] (95% confidence interval [*CI*]) of the Q2, Q3 and Q4 groups were 2.21 (1.85, 2.65), 2.99 (2.51, 3.58) and 4.42 (3.70, 5.27), respectively (trend test *P* < 0.001). Meanwhile, the study divided the participants into two groups based on a cutoff CVAI value of 100 and discovered that the OR value of the high-level group was 2.33 when compared to the low-level group (2.07, 2.62). These findings showed that CVAI and eGFR were negatively correlated (Table 2).

**Table 2:** Multivariate logistic regression analysis of the correlation between CVAI and decreased renal function

CVAI	N	Cases (%)	Crude		Model I		Model II	
			OR (95%CI)	***P value***	OR (95%CI)	***P value***	OR (95%CI)	***P value***
**PerIQR = 59.6**	5355	1858 (34.70)	1.95 (1.80,2.11)	< 0.001	1.82 (1.67,1.98)	< 0.001	1.58 (1.43,1.74)	< 0.001
**Quartiles**								
Q1(≤70.59)	1338	239 (17.90)	1.00 (1.00,1.00)	Ref.	1.00 (1.00,1.00)	Ref.	1.00 (1.00,1.00)	Ref.
Q2(≤101.93)	1339	435 (32.50)	2.21 (1.85,2.65)	< 0.001	2.05 (1.70,2.46)	< 0.001	1.69 (1.38,2.06)	< 0.001
Q3(≤130.23)	1339	528 (39.40)	2.99 (2.51,3.58)	< 0.001	2.64 (2.19,3.17)	< 0.001	2.05 (1.67,2.52)	< 0.001
Q4(≤273.94)	1339	656 (49.00)	4.42 (3.70,5.27)	< 0.001	3.75 (3.10,4.54)	< 0.001	2.77 (2.23,3.44)	< 0.001
Trend test				< 0.001		< 0.001		< 0.001
**CVAI < =100**								
Yes	2589	648 (25.00)	1.00 (1.00,1.00)	Ref.	1.00 (1.00,1.00)	Ref.	1.00 (1.00,1.00)	Ref.
No	2766	1210 (43.70)	2.33 (2.07,2.62)	< 0.001	2.00 (1.76,2.26)	< 0.001	1.64 (1.42,1.89)	< 0.001

*Abbreviation: 95% CI* 95% confidence interval, *OR* odds ratio, *N* numbers of subjects in each stratum, *Case (%)* numbers with 60 ≤ eGFR< 90 and percentage. Model I: adjusted for SBP, FPG, TC, and hs-CRP; Model II: Model I, BUN, uric acid, SCC, SG, CEA, CA211, albumin, and TT3

### CVAI is a good indicator of screening for decreased renal function

**Fig. 2:**
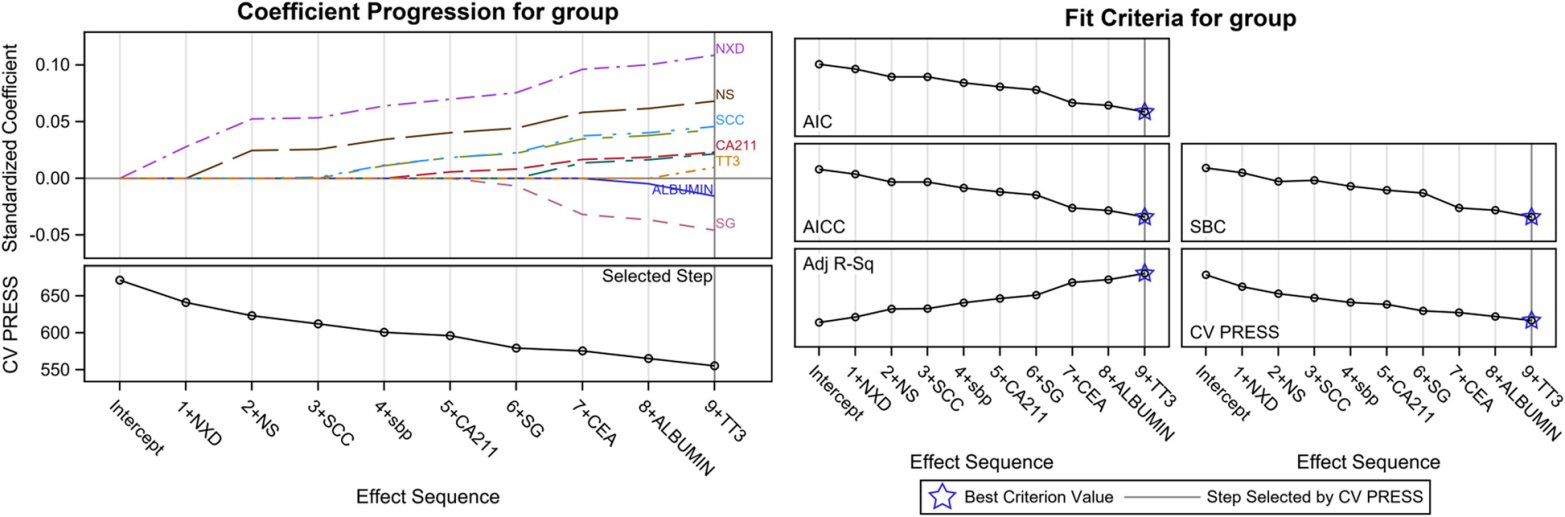
Covariate screening via the LASSO regression algorithm and associated criteria. Abbreviations: BUN: blood urea nitrogen; UA: uric acid; SCC: squamous cell carcinoma; SBP: systolic blood pressure; CA211: carbohydrate antigen 211; SG: specific gravity; CEA: carcinoembryonic antigen; Albumin: serum albumin. TT3: Total triiodothyronine

The CVAI is not the only indicator used to assess renal function. Many other factors are also significantly related to the glomerular filtration rate. Moreover, due to these possible confounding factors, the correlation between CVAI value and eGFR was investigated and corrected for these possible confounding factors (Fig. 2).

Covariate analyses were performed via L1-penalized least absolute shrinkage and selection operator (LASSO) regression and associated criteria. First, the study adjusted several common factors, such as SBP, FPG, TC, and hs-CRP, to obtain model I. The ORs (95% CIs) for Q2, Q3, and Q4 were found to be 2.05 (1.70, 2.46), 2.64 (2.19, 3.17), and 3.75 (3.10, 4.54), respectively. On this basis, the study used LASSO regression to adjust for blood urea nitrogen (BUN), uric acid, SCC, SG, CEA, CA211, albumin, and TT3 and obtained model II values of 1.69 (1.38, 2.06), 2.05 (1.67, 2.52), and 2.77 (2.23, 3.44) for Q2, Q3, and Q4, respectively (Table 2). Moreover, after adjusting for covariates, the risk of renal function injury increased significantly with an increase in CVAI (trend test, *P* < 0.001). Furthermore, the study examined the relationship between CVAI and renal function using a cutoff point of 100. After controlling for the corresponding potential risk factors, the OR values for the high-level groups in models I and II were 2.00 (1.76, 2.26) and 1.64 (1.64, 1.42, 1.89), indicating that CVAI is a good indicator for screening for decreased renal function.

The study also determined whether there was an interaction between some factors affecting renal function (blood pressure, fasting glucose, glycosylated hemoglobin [HbA1c], uric acid, BUN) and CVAI calculations (Table 3). The findings revealed an interaction between uric acid and CVAI, and the effect remained significant after controlling for other confounding variables (*P* < 0.001). Moreover, blood pressure, fasting glucose, and HbA1c showed significant interactions before adjusting for confounding factors (*P* < 0.05).

**Table 3:** Interaction of other factors and point of CVAI for risk of renal function injury

CVAI > 100	Variables	N	Cases (%)	OR (95%CI)	***P value***	OR (95%CI)	***P- value***
	Hypertension						
No	No	2349	551 (23.50)	1.00 (1.00,1.00)	Ref.	1.00 (1.00,1.00)	Ref.
No	Yes	240	97 (40.40)	2.21 (1.68,2.91)	0.000	1.36 (0.96,1.95)	0.087
Yes	No	2108	885 (42.00)	2.36 (2.08,2.69)	0.000	1.72 (1.48,2.01)	0.000
Yes	Yes	658	325 (49.40)	3.19 (2.66,3.81)	0.000	1.71 (1.30,2.24)	0.000
Interaction					0.003		0.082
	FPG > =5.6						
No	No	2398	591 (24.60)	1.00 (1.00,1.00)	Ref.	1.00 (1.00,1.00)	Ref.
No	Yes	191	57 (29.80)	1.30 (0.94,1.80)	0.111	1.05 (0.70,1.56)	0.820
Yes	No	2056	917 (44.60)	2.46 (2.17,2.80)	0.000	1.69 (1.45,1.96)	0.000
Yes	Yes	710	293 (41.30)	2.15 (1.80,2.56)	0.000	1.42 (1.08,1.87)	0.013
Interaction					0.033		0.289
	HbA1c > =5.8						
No	No	2069	453 (21.90)	1.00 (1.00,1.00)	Ref.	1.00 (1.00,1.00)	Ref.
No	Yes	520	195 (37.50)	2.14 (1.74,2.63)	0.000	1.66 (1.32,2.10)	0.000
Yes	No	1485	606 (40.80)	2.46 (2.12,2.85)	0.000	1.67 (1.41,1.98)	0.000
Yes	Yes	1281	604 (47.20)	3.18 (2.74,3.70)	0.000	2.26 (1.86,2.75)	0.000
Interaction					0.000		0.16
	Hyperuricemia						
No	No	2340	533 (22.80)	1.00 (1.00,1.00)	Ref.	1.00 (1.00,1.00)	Ref.
No	Yes	249	115 (46.20)	2.91 (2.23,3.80)	0.000	1.49 (1.05,2.10)	0.024
Yes	No	1994	822 (41.20)	2.38 (2.09,2.71)	0.000	1.84 (1.58,2.15)	0.000
Yes	Yes	772	388 (50.30)	3.43 (2.89,4.06)	0.000	1.38 (1.05,1.81)	0.020
Interaction					0.000		0.000
	BUN> 7.1						
No	No	2475	600 (24.20)	1.00 (1.00,1.00)	Ref.	1.00 (1.00,1.00)	Ref.
No	Yes	114	48 (42.10)	2.27 (1.55,3.33)	0.000	0.68 (0.43,1.07)	0.097
Yes	No	2598	1098 (42.30)	2.29 (2.03,2.58)	0.000	1.62 (1.40,1.87)	0.000
Yes	Yes	168	112 (66.70)	6.25 (4.48,8.73)	0.000	1.36 (0.90,2.06)	0.146
Interaction					0.475		0.445

Hypertension was defined as SBP ≥ 140 mmHg or DBP ≥ 90 mmHg; hyperuricemia was defined as UA > 420 μmol/L in men or UA > 360 μmol/L in women

*Abbreviation: FPG* fasting plasma glucose, *HbA1c* glycosylated hemoglobin, *BUN* blood urea nitrogen

### CVAI has greater value for screening for decreased renal function in women

**Fig. 3:**
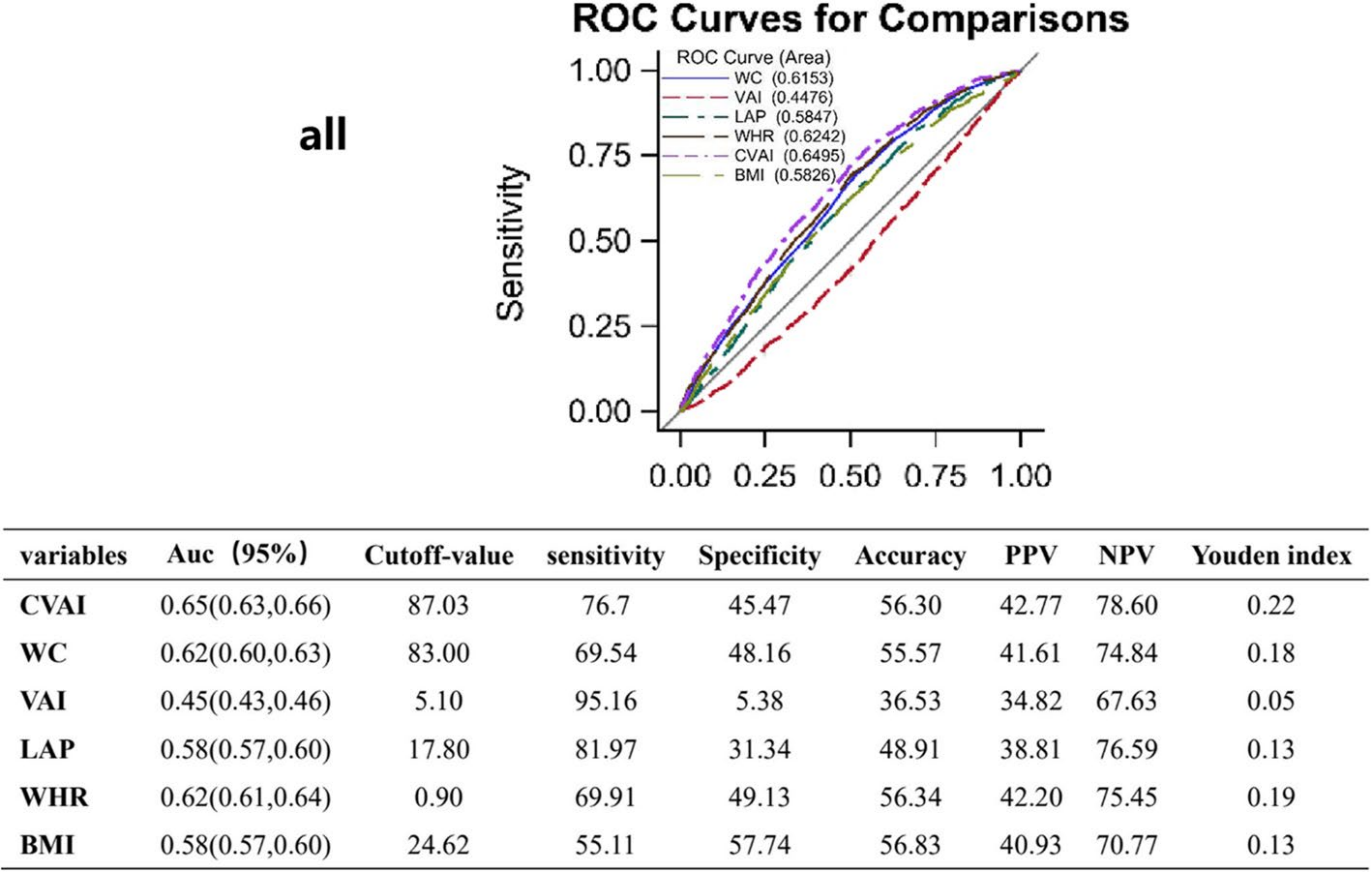
ROC curves for adiposity indices and CVAI point. Abbreviation: CVAI: Chinese visceral adiposity index; WC: waist circumference; VAI: visceral adiposity index; LAP: lipid accumulation product; WHR: waist-to-hip ratio; BMI: body mass index

Based on ROC analysis, the study determined the optimal CVAI cutoff point as the point representing the sum of maximum sensitivity and specificity. Compared to other obesity evaluation indices (BMI, WC, VAI, LAP, and WHR) (Fig. 3), the area under the curve of CVAI was the largest, which was 0.65. (0.63,0.66). Other results included sensitivity (76.70%), specificity (45.47%), positive predictive value (42.77%), negative predictive value (78.60%), Youden index (0.22), and best cutoff value (87.03).

**Fig. 4:**
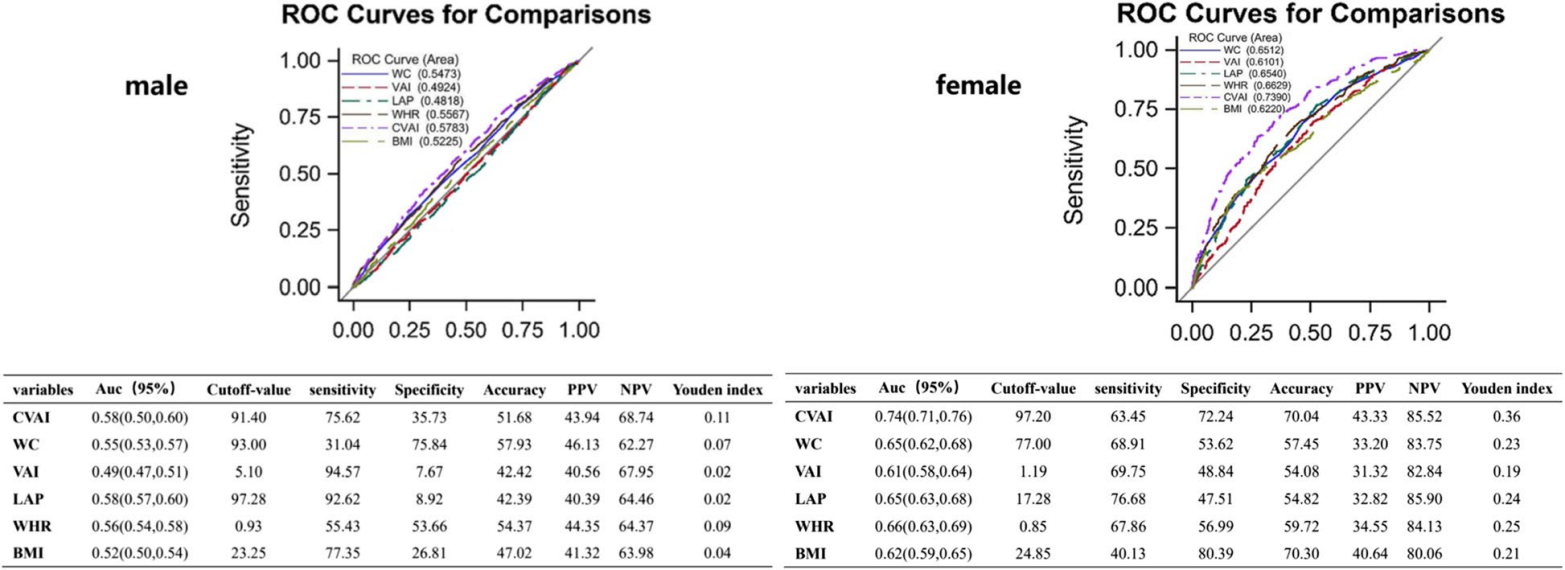
The Obesity Assessment Index (OAI) evaluates the ROC curve of renal function injury based on sex

Considering the effect of sex on fat distribution, this study calculated the area under the ROC curve of CVAI to predict renal function decline according to sex (Fig. 4). which was 0.74 (0.71, 0.76) in the female model, and the Youden index was 0.36, both of which were higher than those of other obesity indicators (BMI, WC, VAI, LAP, WHR). Although the area in the male model decreased [0.58 (0.50, 0.60)], it was still greater than that of BMI, WC, VAI, LAP, and WHR. Through the analysis of the ROC curve, the best cutoff points for males and females were 91.40 and 97.20, respectively.

In addition, to assess the applicability of the results, the study compared the baseline data of the population with decreased renal function into two groups by sex. The study also performed CVAI subgroup analysis based on seven different characteristics [age (< 60, ≥ 60), sex (male and female), BMI (< 24, ≥ 24), hypertension (with or without), FPG (< 5.6, ≥ 5.6), HbA1c (< 5.8%, ≥ 5.8%), and hyperuricemia (with or without)], which revealed that the calculated CVAI value was a good indicator of renal function decline (Table S2, Fig. S2).

## Discussion

The purpose of this study was to explore the correlation between CVAI and renal dysfunction. The study found that CVAI is a simple clinical indicator for assessing obesity, particularly visceral obesity. It can be used to predict the risk of kidney damage after adjusting for various confounding factors. When compared to other obesity indicators (BMI, WHR, WC, LAP, VAI), CVAI shows a promising ability to predict renal function decline, particularly in women. Many factors have been proven to be risk factors for the occurrence of CKD in the past. The study found that age, sex, BMI, blood pressure, fasting blood glucose, HbA1c, and uric acid had a strong relationship with the reduction in renal function, consistent with previous findings [19, 20]. In addition, by combining the covariates screened by LASSO regression, the study evaluated whether there was an interaction between these variables and CVAI. The interaction analysis revealed that blood pressure, HbA1c, uric acid, and CVAI had a significant synergistic effect. Among them, the synergistic effect of uric acid level still existed after adjusting for other variables.

### Comparisons with other studies and what does the current work add to the existing knowledge

Obesity, according to epidemiological studies, is a major risk factor for CKD and can promote the occurrence of kidney diseases [21]. Visceral fat is a major component of fat and has been proven to promote metabolic diseases [13]. Changes in fat composition, such as loss of muscle mass and increased visceral fat, are common in CKD patients [22]. Therefore, exploring the correlation between body fat distribution and CKD is necessary. Clinically, Coutinho discovered that abdominal obesity measures such as WC and WHR are more strongly associated with cardiovascular mortality than generalized obesity measures such as BMI [23]. Furthermore, the obesity phenotype was classified as “metabolic obesity but normal weight” (MONW) or “metabolic health but obesity” (MHO). Azadeh Mottaghi found that MONW patients had a higher risk of CKD [24–26], suggesting that fat distribution is a key factor in preventing CKD. However, because Asians’ fat distribution characteristics differ from those of Europeans and Americans, CVAI, based on Asian body fat distribution characteristics, has a promising application as a new and effective metabolic indicator for visceral fat assessment. It has been widely studied in various disease fields and demonstrated to be an independent predictor for cardiovascular disease, diabetes, and complications [27–29]. Theoretically, it is more appropriate to explain the correlation between visceral fat and CKD in China. This study confirms that CVAI is strongly associated with renal impairment and outperforms other obesity indicators in predicting CKD events.

Interestingly, the study also found that CVAI appears to be more valuable in predicting renal impairment in women, although the mechanism underlying this sex difference is unclear. Different sex hormones may cause differences in fat distribution, explaining the link between obesity phenotypes and chronic kidney disease. Women have higher serum levels of inflammatory cytokines and adipokines than men with the same BMI, which increases the risk of cardiovascular events [30, 31]. Most people included in the study were 45–55 years old; thus, it can be assumed that most women were menopausal or postmenopausal. According to some studies, postmenopausal women accumulate more abdominal fat, and the correlation between visceral fat deposition and insulin resistance is higher than in men; women are thus more likely to develop insulin resistance and type 2 diabetes [32, 33]. Animal studies have also shown that a lack of estrogen and/or estrogen receptors can lead to weight gain, visceral fat increase, and impaired glucose/insulin tolerance [34]. The toxic effects of glucose and lipid accumulation caused by insulin resistance in podocytes and glomerular filtration membranes can accelerate renal injury [35]. This could be one of the reasons CVAI is more predictive of renal function risk in women, but the underlying mechanisms require further investigation.

### Potential biological mechanisms of our findings

Adipose tissue influences the kidney via several secreted factors essential for maintaining normal renal function; however, the specific mechanism is unknown. Some studies have found that in the obese state, adipocytes can undergo hypertrophy or proliferate, macrophages in adipose tissue can transform from the M2 phenotype to the M1 phenotype, and their secretion of inflammatory factors increases, while anti-inflammatory cytokine generation decreases, resulting in systemic chronic inflammation. Nephropathy is associated with the dysregulation of these inflammatory cytokines and adipokines, which work together to cause oxidative stress, inflammation, and fibrotic kidney changes, ultimately leading to kidney damage [36–38]. According to Osama Hamdy, visceral adipose tissue can produce and secrete more proinflammatory cytokines than subcutaneous fat, and visceral fat is the primary cause of systemic inflammation in obesity [39]. Furthermore, visceral adipose tissue can promote insulin resistance, which is stronger than subcutaneous fat [38, 40]. In addition, insulin resistance plays a key role in the development of CKD. It aggravates renal hemodynamics and promotes the progression of kidney disease by stimulating the sympathetic nervous system and the sodium retention system and downregulating the natriuretic peptide system [41]. In addition, adipokines such as leptin also increase the risk of kidney dysfunction. Current data show that leptin levels in CKD patients are elevated due to renal insufficiency, decreased leptin clearance in the renal circulation, and increased leptin secretion from adipose tissue. Leptin production in obese tissue is caused by chronic inflammation, hyperinsulinemia and significant lipid disturbances in CKD patients. Elevated levels of leptin in CKD patients can worsen kidney function and increase cardiovascular risk [42]. This could explain the correlation between visceral adipose tissue and CKD. CVAI combines demography (age), metabolic characteristics (TG and HDL-C), and anthropometry (BMI and WC). CT confirms that it is a reliable indicator in evaluating visceral adipose disorder. As previously stated, these potential mechanisms may explain why individuals with higher CVAI have a higher risk of renal damage in this study.

### Study strengths and limitations

The main advantages of this paper are as follows. The sample size for this population-based study is quite large. To avoid the effects of potential information bias, all laboratory tests were performed strictly according to standard clinical procedures by two well-trained, experienced technicians from the central laboratory of the Second Affiliated Hospital of Zhejiang University School of Medicine. Furthermore, the locally estimated scatterplot smoothing (LOESS) curve was used to describe the relationship between CVAI and eGFR, making the correlation more intuitive and accurate. The LASSO regression algorithm screens covariates reasonably in multivariable regression models, a widely accepted method for achieving appropriate covariates while minimizing potential collinearity in multiple regression. The study used multiple logistic regression to quantitatively describe the correlation between CVAI and eGFR. ROC curves have also been used to determine appropriate thresholds for CVAI to diagnose the occurrence of CKD, which has public health implications.

This study also has some limitations. First, the study was cross-sectional, and we could not establish a causal link between CVAI and decreased renal function. Second, another limitation of this study is the use of post hoc analysis. All CVAI and decreased renal function analyses were conducted in an exploratory manner using existing data. Third, this is a single-center investigation. The research samples were retrieved from the physical examination center of Zhejiang University’s Second Affiliated Hospital; thus, the population may be biased. Fourth, this study excluded people with positive urinary albumin, thereby preventing us from understanding the effect of proteinuria on kidney damage. Fifth, this study does not consider whether these people are candidates for drug treatment or bariatric surgery. This could have impacted the precision of our CVAI calculations. We hope that well-designed future studies will be conducted in the near future to address these limitations. Finally, because MRI and CT imaging tools are expensive and difficult to popularize in large population studies, this study did not accurately evaluate visceral adipose tissue. However, a close relationship between CVAI and visceral adipose tissue has been demonstrated by recent studies.

## Conclusions

In summary, obesity is a major risk factor for CKD, and the risk of renal impairment increases as the CVAI calculated value increases. In all populations, especially in women, the ability of CVAI to diagnose CKD is better than that of VAI, LAP, WHR, WC and BMI, suggesting that CVAI has certain reference value for the screening of CKD patients. Furthermore, it will be useful in guiding us in the primary prevention of CKD.

## Supplementary Information

**Additional file 1: Figure S1.** Flowchart of the selection process of eligible participants. **Table S1.** Sensitivity analysis of some missing variables before and after filling. **Table S2.** Clinical and demographic characteristics of patients with renal function impairment of different sexes. **Figure S2.** Subgroup analysis for the risk of eGFR<90.

## Data Availability

All data used during the study appear in the submitted article.

## Abbreviations

SBP: Systolic blood pressure
BMI: Body mass index
WC: Waist circumference
HC: Hip circumference
WHR: Waist-hip ratio
VAI: Visceral adiposity index
LAP: Lipid accumulation production
CVAI: Chinese visceral adiposity index
CKD: Chronic kidney disease
ESRD: End-stage renal disease
eGFR: Estimated glomerular filtration rate
HDL-c: High-density lipoprotein cholesterol
TG: Triglyceride
TT3: Total triiodothyronine
CA211: Cancer antigen 211
UA: Uric acid
BUN: Blood urea nitrogen
CEA: Carcinoembryonic antigen
SCC: Squamous cell carcinoma
IQR: Interquartile range
OR: Odds ratio
AUC: Area under the curve
MONW: Metabolic obesity but normal weight
MHO: Metabolic health but obesity
