# Supplementary material for: A novel clinical diagnostic marker predicting the relationship between visceral adiposity and renal function evaluated by estimated glomerular filtration rate (eGFR) in the Chinese physical examination population
Source: Lipids Health Dis. 2023 Mar 4;22:32. doi: 10.1186/s12944-023-01783-6 (PMC9985259; doi:10.1186/s12944-023-01783-6)
Supplement: Supplementary file 1 — Additional file 1: Figure S1. Flowchart of the selection process of eligible participants. Table S1. Sensitivity analysis of some missing variables before and after filling. Table S2. Clinical and demographic characteristics of patients with renal function impairment of different sexes. Figure S2. Subgroup analysis for the risk of eGFR<90. [file 12944_2023_1783_MOESM1_ESM.docx]

**Supplementary chart：**


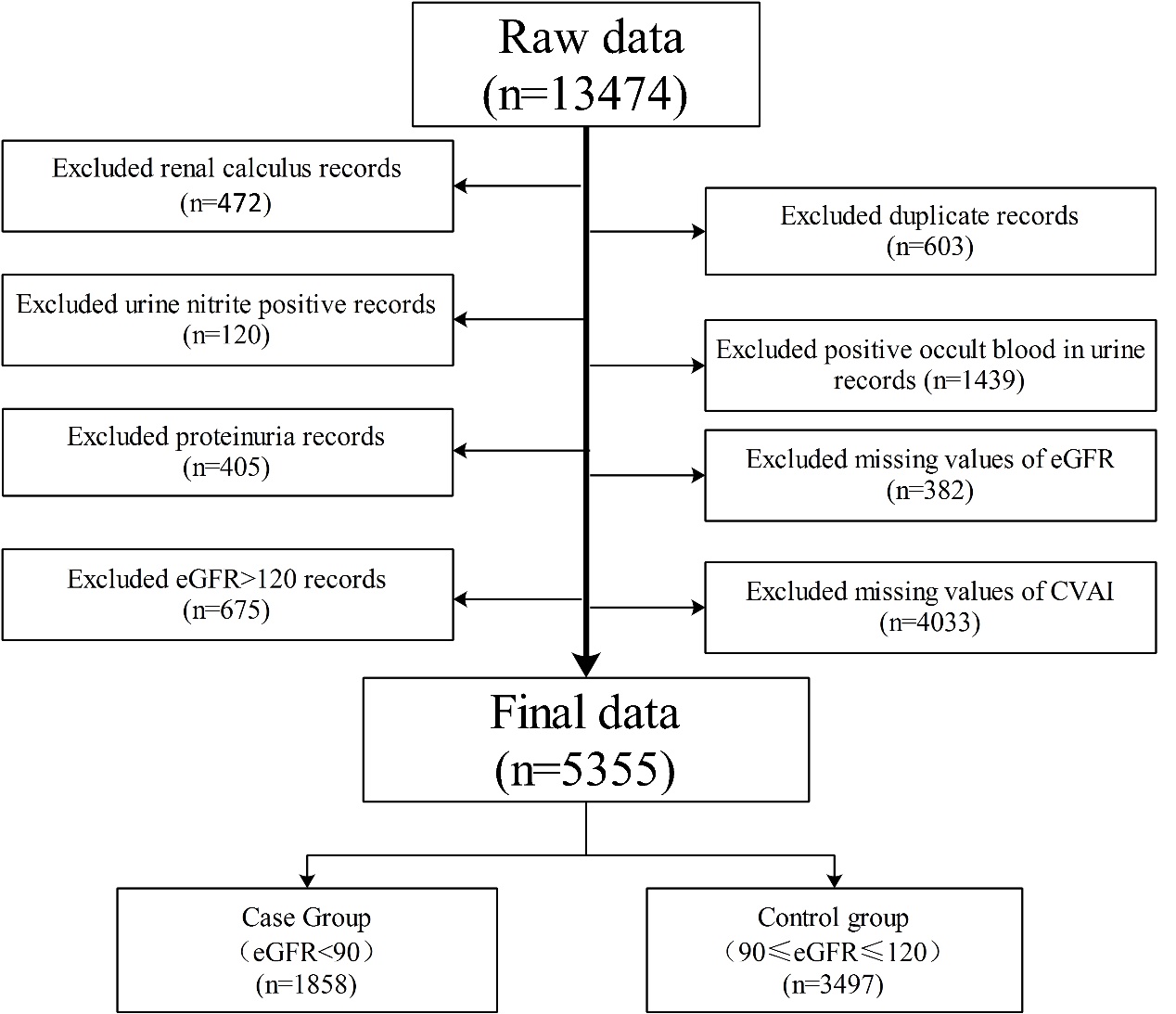


**Figure S1. Flowchart of the selection process of eligible participants.**

***Notes:*** *The process of exclusion is strictly in accordance with the order of the flow chart, and there is no overlap among the exclusion criteria.*

**Table S1 Sensitivity analysis of some missing variables before and after filling**

| **Variables** | **Before filling** | **After filling** | ***P value*** |
| --- | --- | --- | --- |
| **Age (years)** | 51.0(44.6,57.0) | 51.0(44.6,57.0) | 1.000 |
| **Height (cm)** | 166.5(160.0,171.5) | 166.5(160.0,171.5) | 1.000 |
| **Weight（kg）** | 67.0(58.8,75.5) | 67.0(58.8,75.5) | 1.000 |
| **BMI (kg/m2)** | 24.4(22.2,26.5) | 24.4(22.2,26.5) | 1.000 |
| **SBP (mmhg）** | 70(63,79) | 70(63,79) | 1.000 |
| **NBP (mmhg）** | 122(110,134) | 122(110,134) | 0.998 |
| **pulse** | 76(69,83) | 76(69,83) | 1.000 |
| **Albumin (g/L)** | 42.30(40.60,43.90) | 42.30(40.60,43.90) | 1.000 |
| **HbA1c（%）** | 5.70(5.40,6.00) | 5.70(5.40,6.00) | 1.000 |
| **UA（μmol/L）** | 342.00(282.00,403.00) | 342.00(282.00,403.00) | 0.997 |
| **TC（mmol/L）** | 5.12(4.50,5.81) | 5.12(4.50,5.81) | 1.000 |
| **TG（mmol/L）** | 1.34(0.95,1.97) | 1.34(0.95,1.97) | 1.000 |
| **HDL (mmol/L)** | 1.26(1.09,1.48) | 1.26(1.09,1.48) | 0.999 |
| **TT3（ng/ml）** | 1.50(1.35,1.65) | 1.50(1.35,1.65) | 1.000 |
| **hsCRP (mg/L)** | 0.70(0.40,1.50) | 0.70(0.40,1.50) | 0.996 |
| **SG** | 1.02(1.02,1.03) | 1.02(1.02,1.03) | 0.984 |
| **SCC（ug/L）** | 0.80(0.60,1.00) | 0.80(0.70,0.90) | 0.116 |
| **CA211（ug/ml）** | 1.60(1.10,2.20) | 1.60(1.10,2.20) | 1.000 |
| **CEA（ug/ml）** | 1.80(1.20,2.70) | 1.80(1.20,2.70) | 0.999 |

***Abbreviations: BMI:*** *body mass index;* ***DBP:*** *diastolic blood pressure;* ***SBP:*** *systolic blood pressure;* ***FPG:*** *fasting plasma glucose;* ***HbA1c:*** *glycosylated hemoglobin;* ***UA:*** *uric acid;* ***TG:*** *triglyceride;* ***TC: cholestenone; HDL:*** *high-density lipoprotein;* ***LDL:*** *low-density lipoprotein;* ***BUN:*** *blood urea nitrogen;* ***TT3:*** *total triiodothyronine; h****sCRP:*** *hypersensitive C-reactive protein;* ***SG****: specific gravity;* ***CEA:*** *carcinoembryonic antigen;* ***SCC:*** *squamous cell carcinoma*

**Table S2****Clinical and demographic characteristics of patients** **with renal function impairment of different sexes**

| **Variables** | **male** | **female** | ***P value*** |
| --- | --- | --- | --- |
| Age(years) | 56.0(50.0,62.0) | 58.9(54.0,65.0) | <0.001 |
| Height(cm) | 169.5(166.0,173.5) | 156.5(153.0,160.0) | <0.001 |
| Weight(kg) | 73.1±10.2 | 59.0±8.3 | 0.000 |
| BMI(kg/m2) | 25.3(23.4,27.3) | 23.9(21.8,26.1) | <0.001 |
| SBP(mmHg) | 125(115,137) | 127(113,140) | 0.276 |
| DBP(mmHg) | 73(65,81) | 70.00(62,78) | <0.001 |
| FBG(mmol/L) | 4.93(4.59,5.42) | 4.92(4.61,5.29) | 0.339 |
| HbA1c(%) | 5.80(5.50,6.10) | 5.80(5.60,6.10) | 0.058 |
| TC(mmol/L) | 5.08(4.43,5.77) | 5.47(4.77,6.14) | <0.001 |
| TG(mmol/L) | 1.50(1.09,2.21) | 1.23(0.93,1.66) | <0.001 |
| HDL(mmol/L) | 1.16(1.03,1.33) | 1.40(1.24,1.60) | <0.001 |
| LDH(mmol/L) | 2.81(2.33,3.29) | 2.90(2.45,3.44) | 0.001 |
| hsCRP(mg/L) | 1.00(0.50,1.90) | 0.90(0.50,1.75) | 0.419 |
| UA(μmol/L) | 389.50(341.00,440.00) | 301.00(259.00,346.00) | <0.001 |
| BUN(μmol/L) | 5.29(4.59,6.08) | 5.19(4.55,5.93) | 0.094 |
| WC | 88.00(84.00,94.00) | 80.00(75.00,86.00) | <0.001 |
| WHR | 0.94(0.90,0.97) | 0.88(0.84,0.93) | <0.001 |
| LAP | 36.74(22.26,57.76) | 28.35(17.82,42.48) | <0.001 |
| VAI | 1.67(1.10,2.64) | 1.62(1.07,2.36) | 0.046 |
| CVAI | 117.17±38.77 | 108.96±40.06 | 0.000 |

*Abbreviations:* ***BMI:*** *body mass index;* ***DBP:*** *diastolic blood pressure;* ***SBP:*** *systolic blood pressure;* ***FPG:*** *fasting plasma glucose;* ***HbA1c:*** *glycosylated hemoglobin;* ***TG:*** *triglyceride;* ***TC*:** *cholestenone****; HDL****: high-density lipoprotein;* ***LDL:*** *low-density lipoprotein; h****s-CRP:*** *hypersensitive C-reactive protein;* ***UA****: uric acid;* ***BUN:*** *blood urea nitrogen;* ***WC****: waist circumference*; ***WHR***: *waist-hip ratio;* ***VAI:*** *visceral adiposity index*; ***LAP:*** *lipid accumulation production;* ***CVAI***: *Chinese visceral obesity index*


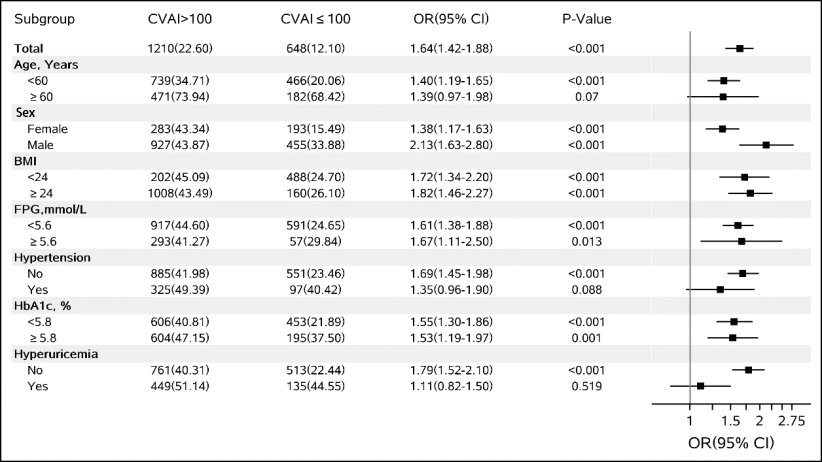


**Figure S2 Subgroup analysis for the risk of eGFR<90**

***NOTES:*** *Hypertension was defined as SBP≥140 mmHg or DBP≥90 mmHg; hyperuricemia was defined as UA>420 μmol/L in men or UA>360 in women; Abbreviations: BMI: body mass index; FPG;fasting plasma glucose HbA1c: glycosylated hemoglobin;*
